# Zero-shot personalization of speech foundation models for depressed mood monitoring

**DOI:** 10.1016/j.patter.2023.100873

**Published:** 2023-11-01

**Authors:** Maurice Gerczuk, Andreas Triantafyllopoulos, Shahin Amiriparian, Alexander Kathan, Jonathan Bauer, Matthias Berking, Björn W. Schuller

**Affiliations:** 1 Chair of Embedded Intelligence for Healthcare and Wellbeing, University of Augsburg, Augsburg, Germany; 2 Department of Clinical Psychology and Psychotherapy, Friedrich-Alexander-Universität, Erlangen-Nürnberg, Erlangen, Germany; 3 GLAM, Imperial College, London, UK

**Keywords:** personalization, depression monitoring, foundation models, speech processing, hypernetworks, deep learning

## Abstract

The monitoring of depressed mood plays an important role as a diagnostic tool in psychotherapy. An automated analysis of speech can provide a non-invasive measurement of a patient’s affective state. While speech has been shown to be a useful biomarker for depression, existing approaches mostly build population-level models that aim to predict each individual’s diagnosis as a (mostly) static property. Because of inter-individual differences in symptomatology and mood regulation behaviors, these approaches are ill-suited to detect smaller temporal variations in depressed mood. We address this issue by introducing a zero-shot personalization of large speech foundation models. Compared with other personalization strategies, our work does not require labeled speech samples for enrollment. Instead, the approach makes use of adapters conditioned on subject-specific metadata. On a longitudinal dataset, we show that the method improves performance compared with a set of suitable baselines. Finally, applying our personalization strategy improves individual-level fairness.

## Highlights

• We study a longitudinal automatic recognition of depressed mood from speech
• We propose a metadata-based personalization of large speech foundation models
• Our approach shows substantial per-speaker performance improvements

## The bigger picture

Depression, as one of the most prevalent mental health diseases, negatively impacts millions of lives. Diagnoses are achieved by the assessment of symptoms with standardized tests. However, recent studies indicate that continuously monitoring symptoms (e.g., with ecological momentary assessments [EMAs]) may provide relevant additional information for both diagnosis and treatment decisions. More recently, these manual methods have been complemented by passive sensing methods. Here, speech can serve as a valuable objective marker because it has been shown to be impacted by various pathologies, such as anxiety and mood disorders, and can be collected non-invasively and cheaply. Existing machine learning methods that aim to measure mood, however, often fail to accurately model intra-individual variations, assuming that data are sourced from homogeneous populations. We introduce and evaluate an effective zero-shot personalization of speech foundation models that utilizes diagnostic information about each patient to improve per-speaker depressive mood recognition over a 2-week EMA period.

## Teaser

Gerczuk et al. propose a zero-shot personalization strategy of large speech foundation models for the automatic recognition of depressed mood. The approach works solely based on patient metadata and is shown to substantially improve per-speaker performance on a longitudinal dataset of speech recordings and associated mood ratings.

## Introduction

Major depressive disorder is one of the most prevalent mental health diseases, afflicting millions around the world and having severe repercussions for the quality of life on an individual and a societal level.^1^^,^^2^ Its manifestation covers a wide gamut of symptoms,^3^ with depressive mood being a core one.^4^ This emerges as a by-product of emotion dysregulation caused by depression-induced cognitive biases, which are involved in the development and persistence of depression.^5^ Symptom monitoring allows the timely characterization of disease status or detection of relapse and plays a key role in facilitating individualized treatment plans.^6^ In particular, depressed mood has been found to be a good indicator of treatment response, being one of the first symptoms to improve.^7^ In addition, psychotherapy patients are often required to monitor their mood over the course of treatment,^8^ making it one of the most widely used diagnostic means. This is typically done in the form of ecological momentary assessments (EMAs), where patients are required to fill in standardized questionnaires at regular intervals.

In recent years, these manual EMAs are often complemented by non-invasive, passive sensing methods.^9^^,^^10^ Typically, these take the form of mobile monitoring applications that utilize various sensors embedded in modern smartphones or wearables and have been shown to correlate with mental states.^10^^,^^11^^,^^12^ Speech, as one of the biomarkers affected by different pathologies,^13^^,^^14^^,^^15^ such as mood disorders^16^ and depression,^17^^,^^18^^,^^19^ can be used as a means to passively monitor patients. This can be done through the pervasive recording of daily life,^20^ using minimalistic models deployed in edge devices,^21^ monitoring telephone conversations,^22^ or eliciting responses through human-computer interaction interfaces (e.g., computer games^23^) in a naturalistic setting. In this way, high-dimensional speech patterns can be obtained and their changes analyzed in real time, allowing clinicians to tailor interventions to an individual’s specific needs, optimizing the effectiveness of therapy and medication management.^24^

Recent works also pursue a confluence between manual EMAs and automated analysis methods. Notably, voice-based interfaces present a natural way to elicit information about a patient’s mood. This can form a complementary source of information alongside traditional EMAs and provide a deeper understanding of the patients’ mood and emotions.^25^ Here, speech has been shown to not only capture affective^26^ but a wider range of speaker states, such as intoxication,^27^ which could have distorting effects on the expression and experience of mood.^28^ Furthermore, while anxiety and depression negatively impact mood, how exactly they are reflected in a single self-rated mood item can differ between individuals based on arousal and valence focus.^29^^,^^30^ Because a variety of its characteristics are associated with anxiety levels,^31^ speech can help to untangle and explain these influencing factors.

Speech can thus form a complementary lens through which to analyze patient responses to EMAs, with the potential to uncover more insights and provide a more holistic understanding of how depressive mood changes over time; e.g., through a post hoc interpretation of the features most associated with mood prediction.^32^

Passive monitoring and a more holistic characterization of depression therefore form the key promises of speech-based analysis. Both begin from a well-performing predictive model for depressive mood, which can either be used in a passive setup or interpreted to provide the required insights. However, existing modeling approaches operate under the assumption that the data are sourced from a homogeneous population, thus failing to capitalize on individual differences across patients. This is often crucial in depression monitoring, where population-level models may not generalize adequately to individual depression scores.^33^ Prior works have already exploited personalized models to predict daily depression mood using speech data.^25^^,^^34^ These works rely on patient-specific subnetworks, with individual-level output heads trained on top of a population-level core.^35^ Crucially, these works rely on an enrollment phase, where patients are required to provide a series of labeled speech data for training the system. Furthermore, these data need to be provided already at the training phase and do not allow post hoc adaptation to new patients.

An alternative approach to personalization can be achieved through the use of patient-specific metadata. These metadata can be in the form of demographic information (such as gender or age), previous history (e.g., diagnostic tests or medication), or depression monitoring scales that are collected as part of their routine monitoring. These rich metadata form an additional, often underutilized, source of information that can condition a predictive model.

In the present work, we propose a method for speech-based, personalized depressed mood prediction. Our method achieves a multistage fusion of personal metadata with elicited speech responses by co-opting hyperformer adapters, a recently introduced approach for facilitating more rapid adaptation to new tasks for transformer-based models.^36^^,^^37^ These adapters are trainable modules that are appended to the output of each intermediate layer, forming a means with which to inject new information. This information primarily denotes the downstream task on which the model is expected to generalize. To the best of our knowledge, this mechanism has not been previously used for personalization.

Besides our method, we place additional emphasis on evaluating our results. Importantly, we go beyond standardized, population-level evaluations and include comprehensive, individual-level metrics. These provide us with a more granular view of model performance and behavior, which enables us to gauge the positive impact of personalization and further promotes recent attempts to measure individual-level performance.^12^

## Results

We first present the dataset utilized in our study, including a short exploratory data analysis, giving some insights into the relationship between acoustic features and depressed mood. Because our personalization approach for mood monitoring relies on the injection of diagnostic meta-information, such as scores from depression questionnaires, we refrain from performing a speech-based analysis of these factors. Instead, we refer the interested reader to Cummins et al.^18^ for the influence of a range of acoustic features on patient health questionnaires (PHQs) and to He et al.^38^ for an overview of deep learning approaches for depression recognition. Moreover, we briefly look at the relationship between diagnostic scores and self-assessed depressed mood. Afterward, a structured analysis of experimental results follows. Apart from comparing our proposed zero-shot personalization with the implemented baselines, we further highlight the influence of recorded speech content as well as ablating the model performance with regard to the choice of embedded meta-information. For these results, we focus on analyzing per-individual performance measured in the mean of per-speaker Spearman’s ρ, calculated for each of the five folds in a speaker-independent cross-validation and then averaged. We briefly discuss global performance and why it is ill-suited to gauge the efficacy of personalization in the chosen methodology. We report metrics for all subjects (all) and for each of the three subject groups (control, subclinical, and patients) separately. Finally, we analyze the fairness of the personalized models compared with our baseline.

### Dataset

We utilize a longitudinal dataset of speech recordings collected as part of the multistage study in the DFG (German Research Fund) project ParaSpeChaD. The project was approved by the ethics committee of the Friedrich-Alexander-Universität, Erlangen-Nürnberg, Germany. Written informed consent for the scientific processing of recorded data and publishing of experimental results was obtained from all participants. It includes data from a total of 143 subjects (48 male [m], 95 female [f], and one with diverse gender) aged between 18 and 63 years (mean 32.7 and standard deviation 11.0 years). All subjects were native German speakers. To ensure variability in the severity of depressive symptoms and to prevent systematic distortions because of gender and age, participants were distributed into three experimental groups based on their PHQ-9 and current diagnosis of major depressive disorder (MDD) during pre-screening. This resulted in the inclusion of 47 healthy controls (control, PHQ-9 ≤4), 48 subclinical individuals (subclinical, PHQ-9 >4), and 48 subjects afflicted with MDD measured by the Structured Clinical Interview for DSM-5 (SCID-5) (patients). A chi-square test showed no statistical dependence between experimental group and gender: $\chi^{2}\left(2,N=143\right)=0.21$, p=0.99. Furthermore, a one-way ANOVA was used to confirm that there was no significant difference in mean age between the experimental groups (p=0.97).

For the purposes of the evaluated machine learning experiments, we created a speaker-independent 5-fold cross-validation. Inside each of these folds, we further split a fifth of speakers from the training set into a validation partition utilized for early stopping and choosing final model checkpoints.

#### Speech recordings and mood ratings

**Figure 1:**
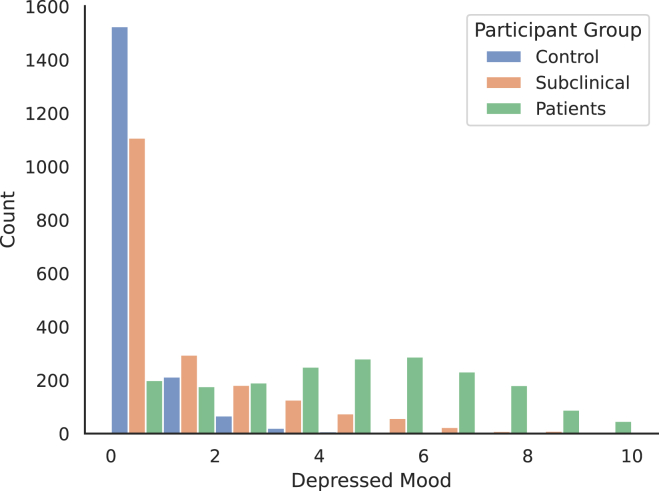
Distribution of depressed mood Distribution of self-assessed depressed mood in the study groups.

After screening and baseline assessments, participants underwent a 2-week EMA via a smartphone application. Three times a day (morning, noon, and evening), the application notified them to perform a short recording session and self-assess their current depressed mood. The recordings consisted of reading out loud and answering three mood-related questions: “How are you feeling right now?”, “How are you coping with this feeling?”, and “How do you plan to deal with this feeling?”. Afterward, a spontaneous positive thought should be formulated and recorded three times in a row. At the end of the session, participants rated their current depressed mood on the discrete visual analog mood scale (VAMS)^39^ from 0 (not depressed) to 10 (severely depressed). In total, each participant, therefore, recorded longitudinal data with a maximum of (3+3+3)×(3)×(14)=378 speech samples and 42 associated mood ratings. As expected for the EMA applied in our study, however, some recording sessions were missed or skipped by participants, reducing the maximum of 54,054 audio samples recorded in 6,006 sessions to 50,779 speech recordings obtained in 5,660 sessions. The number of missed sessions differs between the three groups of participants, roughly increasing with the diagnosis of depressive symptoms. In the healthy control group, 169 sessions are missing, while in the subclinical and patient groups, 200 and 233 recording sessions were skipped, respectively. On the other hand, some participants recorded more sessions than asked for. Finally, mood ratings are distributed differently across the participant groups, as seen in Figure 1. Healthy controls exhibit the most minor variation, mostly assessing their depressed mood as minimal (0). The ratings in the subclinical group are similarly skewed toward the minimum of the scale but extend farther toward higher depressed mood. For subjects in the patient group, mood is more uniformly distributed around the center of the scale but with a heavier tail at the lower end.

#### Available demographic and diagnostic information

In addition to the recordings and mood ratings collected in the EMA, which form the target data for our machine learning analysis, an assortment of metadata about each study participant is available, ranging from demographic information to depression interviews and questionnaires. These data were recorded either during pre-screening (mainly demographics) or later in a baseline assessment directly before the EMA period. We chose a subset of this information to incorporate into our zero-shot personalization strategy, consisting of some demographic information, an assortment of self- and therapist-rated depression questionnaires, a questionnaire concerned with emotional competence, and a dimensional personality trait measurement.

Table 1 contains a complete account of utilized patient-specific meta-information. For demographics, we chose to include age, gender, school and professional degrees, and employment status. We code these attributes either as continuous (age), ordinal (level of degrees), or categorical (gender, employment status) values. Furthermore, we include whether participants are currently taking any medication that could affect either their mood or their voice.

**Table 1:** Description of subject-specific metadata available in our dataset

Metadata	Description	Values
Demographic information		

Age	age at start of study	[18,63]
Gender	categorical male/female/diverse	{0,1,2}
School degree	increasing levels of the German tripartite school system (none, “Mittelschule” (general), “Realschule” (more practical), “Abitur” (more academic)	{0,..,3}
Professional degree	increasing (none, studying, vocational degree, university degree)	{0,..,3}
Full-time job	Binary	{0,1}

Medication		

Medication affecting voice	binary: whether the subject is currently taking medication that could affect voice quality	{0,1}
Medication affecting mood	binary: whether the subject is currently taking medication that could affect mood	{0,1}

Depression tests		

HRSD-24	clinician-rated depression interview with items rated on 3- to 5-point scales	${\left\{0,..,4\right\}}^{24}$
PHQ-9	self-assessed depression questionnaire with items rated on 4-point scales	${\left\{0,..,3\right\}}^{9}$
BDI	self-assessed depression questionnaire with items rated on 4-point scales	${\left\{0,..,3\right\}}^{21}$

Other tests		

SEK-27	self-assessed questionnaire measuring emotional competence via 27 items	${\left\{0,..,4\right\}}^{27}$
TIPI	self-assessed questionnaire measuring Big Five personality dimensions via 10 items	${\left\{0,..,6\right\}}^{10}$

We utilize vector representations of different subsets for personalization.

The Hamilton Rating Scale for Depression (HRSD),^40^ the PHQ-9,^41^ and the Beck’s Depression Inventory (BDI)^42^ form our set of depression tests, providing information about a participant’s symptomatology, measured just before the EMA phase. The 24-item version of the HRSD was obtained through a clinical interview, conducted in the baseline assessment, and is the only of the three depression tests that is not self-rated through a questionnaire. We encode the scores of each item as continuous variables (ranging from 0–5). For the PHQ, we choose the 9-item questionnaire, with each item rated on a 4-point scale. While the BDI, which aims to measure the severity of depressive symptoms, is only validated for depressed individuals, we still include it for personalization in all participant groups, with non-patients rating most items at 0.

Additionally, we include the Self-Report Measure for the Assessment of Emotion Regulation Skills (SEK),^43^ a 27-item questionnaire that, compared with depression assessment scales, focuses entirely on emotion regulation capabilities. It measures 9 dimensions relevant to the constructive handling of negative emotions as a trait as well as a prolonged state. Higher scores in these items represent more effective regulation skills. Finally, the Ten-item Personality Inventory (TIPI)^44^ serves as a short measurement of the different dimensions in the Big-Five framework of personality traits. Its 10 Likert items are self-rated by subjects on 7-point scales.

For the purpose of evaluating the results of our proposed metadata-based personalization strategy, it is important to note that the diagnostic metadata are highly informative of inter-subject differences in depressed mood; e.g., a high PHQ-9 score will, on average, be reflected in a higher depressed mood. The first row of Table 3 shows the Spearman’s rank correlations of depressed mood ratings and total scores of PHQ-9, HRSD, and SEK. Our evaluation therefore focuses on how models are able to recognize mood changes measured in mean per-speaker correlations between model predictions and ground truth ratings.

### Exploratory data analysis

We present an exploratory data analysis of the impact of depressed mood on the acoustic features fundamental frequency (*F0*), harmonic-to-noise ratio (HNR), local jitter and shimmer, number of syllables (*nsyll*) and pauses (*npause*), and duration, as well as speech rate (extracted with Praat^45^ using its Python wrapper Parselmouth^46^), which are known from the literature to correlate with depression.^47^^,^^48^ Rather than an exhaustive study, this helps to understand our dataset and serves as a motivating primer for the influence of personalized metadata on the features of different patients. For a comprehensive overview of the impact of depression on features, see Cummins et al.^19^

**Figure 2:**
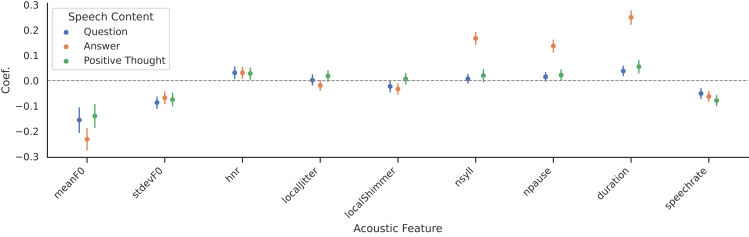
Relationship between acoustic features and mood LMM coefficients (with 95% confidence intervals) of acoustic features for self-assessed depressed mood, grouped by speech content.

Figure 2 depicts the linear mixed-effect model (LMM) coefficients of acoustic features for depressed mood rated by self-assessment, grouped by subject. Acoustic features are normalized to zero mean and unit variance, and we include a random intercept to account for inter-subject differences. The y axis represents the change in self-perceived depressed mood (scale 0−10) associated with an increase of one standard deviation in the respective acoustic feature. In our analysis, we distinguish between three speech contents: question, answer, and positive thought.

In our dataset, it can be observed that *F0*, *nsyll*, and *npause* in an utterance and the overall duration have an impact on depressed mood. Previous research showed that increasing symptom severity goes along with a reduced *F0*,^48^^,^^49^ typically observed in people suffering from depression. Moreover, Wang et al.^49^ analyzed the effect of a clinical intervention on the acoustic characteristics of study participants. In doing so, they concluded that there is a correlation between *F0* and depression and that *F0* increases after successful treatment. Figure 2 confirms that there is a negative correlation between the depressed mood and *F0*, which means that people with a very depressed mood exhibit a lower *F0* compared with people with a less depressed mood.

Furthermore, it can be observed that patients with increasing depressed mood speak longer on average. The greater duration is due to a longer response (positive coefficient for *nsyll*) as well as an increased number of pauses during speaking (positive coefficient for *npause*) compared with less depressed people. The negative association of mood with speech rate strengthens the observation that depressed people speak longer but slower, a typical feature of depression explored in previous work. For example, Cannizzaro et al.^47^ and Cummins et al.^48^ showed that increased speech pause time (i.e., slower speech rate) and total speech time are associated with higher scores on the HRSD and other scales of psychomotor speed and subject self-rating of mood.

Another insight is gained by comparing the coefficients broken down by speech content. The effect sizes are highest when using the speech content “answer,” whereas “question” and “positive thought” usually show lower associations with depressed mood (cf. Figure 2). The only exception is *F0*. However, considering that the question is a predefined text, it seems reasonable that it correlates with *F0* but less strongly with other features, such as duration. In contrast, patients can answer freely, which is why a higher impact of speech duration is to be expected. “Positive thought,” on the other hand, were also freely formulated and show lower coefficients as well. One hypothesis to explain this would be that the intervention (“formulate a positive thought”) might, on average, push people away from their current (bad) mood, which, in turn, makes it harder for the model to detect depression.

Overall, we can conclude in our EDA that depressed people speak slower (and therefore with a longer duration), make more pauses, and exhibit a lower *F0* compared with less depressed people, which is also confirmed in previous research. In addition, the type of speech content has a great influence on the correlation with depression. To this end, speech content in which people speak freely, such as the answers, works best.

**Figure 3:**
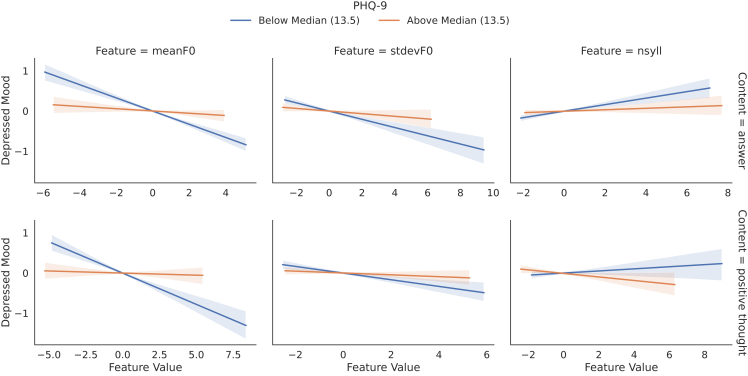
Interaction between PHQ-9 and expression of mood in speech Interactions of PHQ-9 scores in the patient group with the influence of acoustic features on self-rated depressed mood. Speaker-standardized feature values and self-assessed depressiveness are plotted against each other, separated by median PHQ-9 scores.

Previous studies have shown that different groups of participants can have different feature characteristics.^50^ To further analyze the influence of acoustic features on different groups of people and their self-rated depressed mood, we divide them into participants with a PHQ-9 above and below the median value (13.5), respectively, providing insights into the potential of personalized approaches. We further normalize the depressed mood ratings as well as the acoustic features for each speaker separately. Figure 3 shows that participants with a lower PHQ-9 score (below median PHQ-9) have a higher correlation for three exemplary speech features with self-rated depressed mood compared with participants with a higher PHQ-9 score (above median PHQ-9). One hypothesis to explain this observation would be that less depressed people exhibit a greater variation in their speech characteristics. In contrast, participants with a higher PHQ-9 value seem to be more consistent and show less speech variation with changing depressed mood. An alternative hypothesis would be that high-PHQ-9 people show less variation in their mood rather than their characteristics; i.e., they are most often in a depressed mood irrespective of their tone of voice.

The greatest differences among the two groups (above and below median) can be found for mean *F0*, one of the features with the highest correlation in Figure 2. If the influence of the acoustic features on self-rated depressed mood is further subdivided for the two groups according to speech content (“answer” and “positive thought”), it appears that both speech contents show a similar trend. For the sake of completeness, it should be mentioned that there are hardly any differences between the two groups below and above median PHQ-9 using “question” as speech content (not depicted in Figure 3). However, this might be explained due to “question” being one of the groups with the lowest correlations (cf. Figure 2) and questions being pre-defined, resulting in less speech variation between individuals.

In summary, our analyses show that, while there are acoustic markers for depressed mood, there can be considerable differences in the feature expression between different groups of people, which poses a challenge for machine learning algorithms trained on an overall population. In the case of our data, diagnostic meta-information, such as the severity of depression, seem to interact with the relationship between the acoustics of a person’s speech and their self-assessed depressed mood. These findings form the motivation for our proposed personalization strategy, which conditions speech foundation models on learned embeddings of a subject’s metadata.

### Performance of the proposed approach

Table 2 shows the results achieved with the proposed zero-shot personalization strategy based on hyperformers (details can be found under Zero-shot personalization through metadata-conditioned hyperformers) evaluated against a set of baselines, described under Baselines. We report the mean absolute error (MAE) between model predictions and ground truth—showing how closely model predictions align with the subjective mood ratings—as well as the mean of per-speaker correlations (Spearman’s ρ), which indicate how accurate the models are in predicting changes in depressed mood. Before delving into the results, we should again stress that global correlations computed over predictions across different speakers do not provide any insights about performance because of the nature of the dataset and personalization strategy. Table 3 shows correlations of the model outputs with ground truth mood ratings and the scores of PHQ, HRSD, and SEK. Furthermore, we provide correlations of the ground truth and these scores. It is apparent that, globally, the mood ratings are moderately to strongly correlated with the scores. Because the subject embeddings used for personalization are formed from these scores, inter-individual differences in depressed mood can be inferred quite reliably. This is further supported by the fact that the output of all personalized models correlates more strongly with the scores from the questionnaires than the target ground truth mood ratings.

**Table 2:** Speech-based results (mean and SD over 5 folds)

	All		Control		Subclinical		Patients	
	MAE	Speaker ρ	MAE	Speaker ρ	MAE	Speaker ρ	MAE	Speaker ρ
Baseline eGeMAPS FFNN	1.873±0.109	0.132±0.040	1.375±0.131	0.100±0.031	1.461±0.192	0.133±0.092	2.770±0.411	0.157±0.070
Baseline wav2vec FFNN	1.826±0.182	0.175±0.091	1.323±0.427	0.143±0.118	1.578±0.223	0.186±0.105	2.525±0.315	0.181±0.087
Fine-tuned wav2vec	1.757±0.163	0.299±0.076	1.095±0.189	0.225±0.089	1.413±0.244	0.325±0.116	2.737±0.457	0.329±0.077
Personalized eGeMAPS FFNN	0.981±0.097	0.143±0.056	0.298±0.057	0.129±0.053	0.828±0.163	0.144±0.089	1.765±0.244	0.143±0.073
Personalized wav2vec FFNN	1.038±0.154	0.138±0.043	0.392±0.048	0.159±0.043	0.896±0.244	0.127±0.070	1.758±0.213	0.125±0.058
Hyperformer all	1.186±0.177	$0.373^{*}\pm 0.045$	0.396±0.076	0.319±0.069	0.999±0.147	$0.370^{*}\pm 0.061$	2.096±0.376	$0.422^{*}\pm 0.069$
Hyperformer PHQ-9	1.210±0.163.	0.358±0.050	0.418±0.077	0.318±0.077	0.975±0.119	0.338±0.083	2.170±0.349	0.414±0.074
Hyperformer HRSD	1.241±0.165.	0.343±0.060	0.394±0.091	0.258±0.093	0.957±0.145	0.350±0.074	2.295±0.389	0.405±0.056
Hyperformer BDI	1.241±0.176	0.362±0.053	0.376±0.093	0.339±0.085	1.038±0.180	0.351±0.081	2.236±0.362	0.395±0.062
Hyperformer depression tests	1.190±0.146	0.371±0.069	0.330±0.088	$0.341^{*}\pm 0.114$	0.955±0.155	0.354±0.092	2.209±0.335	0.420±0.082
Hyperformer demographics + medication	1.393±0.199	0.325±0.070	0.498±0.145	0.257±0.082	1.167±0.193	0.324±0.108	2.438±0.516	0.370±0.085
Hyperformer SEK	1.280±0.205	0.355±0.049	0.489±0.093	0.289±0.092	1.025±0.176	0.347±0.060	2.267±0.409	0.407±0.066
Hyperformer personality	1.314±0.202	0.353±0.075	0.420±0.097	0.253±0.081	1.064±0.189	0.369±0.084	2.386±0.449	0.413±.097

MAE takes all model predictions per fold into account, while Spearman’s ρ is computed for each speaker independently and then averaged over all speakers. The best results per subject group are marked by an asterisk (∗).

**Table 3:** Spearman’s correlations between model predictions of depressed mood, ground truth self-ratings, and score sums of PHQ-9, HRSD-24, and SEK

Experiment	Spearman’s correlation (ρ)			
	PHQ	HRSD	SEK	Ground truth
Ground truth	0.694±0.075	0.683±0.069	0.630±0.045	1.000±0.000
Baseline eGeMAPS FFNN	0.140±0.130	0.120±0.148	0.174±0.075	0.161±0.081
Personalized eGeMAPS FFNN	0.878±0.064	0.839±0.029	0.836±0.069	0.737±0.051
Baseline wav2vec FFNN	0.287±0.142	0.272±0.150	0.325±0.161	0.314±0.118
Personalized wav2vec FFNN	0.873±0.062	0.841±0.026	0.788±0.051	0.734±0.054
Fine-tuned wav2vec	0.261±0.103	0.253±0.096	0.306±0.108	0.315±0.077
Hyperformer all	0.731±0.116	0.741±0.073	0.679±0.076	0.709±0.066
Hyperformer BDI	0.682±0.114	0.665±0.063	0.641±0.061	0.668±0.063
Hyperformer demographics + medication	0.529±0.127	0.584±0.104	0.498±0.140	0.562±0.041
Hyperformer depression tests	0.739±0.082	0.750±0.060	0.678±0.067	0.700±0.075
Hyperformer HRSD	0.656±0.102	0.708±0.094	0.623±0.057	0.663±0.059
Hyperformer PHQ-9	0.759±0.081	0.671±0.060	0.676±0.051	0.686±0.062
Hyperformer personality	0.689±0.057	0.613±0.064	0.588±0.114	0.642±0.043
Hyperformer SEK	0.658±0.120	0.616±0.087	0.681±0.103	0.644±0.044

Computed globally for each fold and then averaged.

Looking at Table 2, it is immediately evident that our approach improves performance compared with the non-personalized baseline models. From the baselines, the feedforward neural networks (FFNNs) perform worst, only reaching a low 0.175 mean per-speaker Spearman correlation, while fine-tuning the transformer encoder of a pre-trained wav2vec model lands at 0.299. For the proposed personalization strategy, on the other hand, conditioning the hypernetworks on a subject embedding generated from all demographic and diagnostic metadata (Hyperformer All in the table) described under Available demographic and diagnostic information lowers the globally computed MAE from 1.757 to 1.186 and increases the mean of per-speaker correlations from 0.299 to 0.373 when compared against the fine-tuned wav2vec baseline.

**Figure 4:**
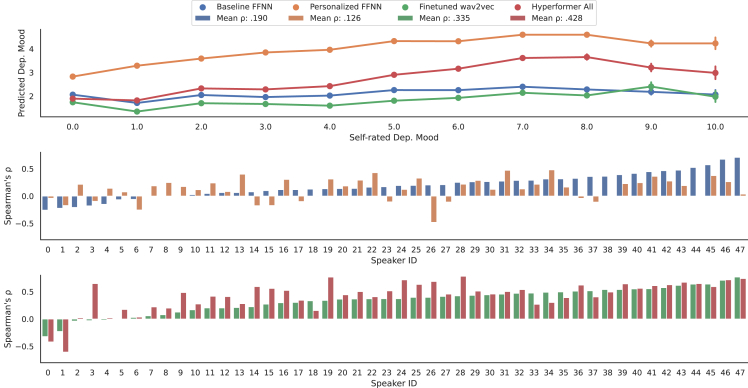
Global vs. speaker-level performance evaluation Global and speaker-level performance implications (measured in Spearman’s ρ) of our proposed zero-shot personalization of transformers compared with a simple metadata personalization. While both personalization strategies improve the predictive performance globally (top plot) compared with their respective baselines, only the proposed Hyperformer approach (bottom plot) improves per-speaker performance in the majority of cases. The top plot shows the distribution of model predictions per distinct value on the ground truth rating scale (integers from 0–10). The bottom plots compare speaker-level Spearman’s ρ of the two personalization strategies and their respective non-personalized baselines. Speakers are sorted by baseline performance. We additionally report the mean of per-speaker Spearman’s ρ for each model.

In the case of the simple personalized baseline FFNNs, where the outputs of the neural network backbones are additively adjusted by the projected and embedded metadata vectors, the personalized FFNN trained on the extended Geneva minimalistic acoustic parameter set (eGeMAPS) of audio functionals achieves the best global performance of 0.981 MAE, while per-speaker ρ sees barely any improvement from its non-personalized counterpart (0.143 vs. 0.132). A similar trend can be seen when choosing wav2vec as input features to the FFNN. We suspect that the personalized model mainly shifts its predictions based on the severity of each person’s depression but is not able to improve intra-subject mood recognition. Our suspicion is confirmed by contrasting the global distribution of model predictions in the patient group against speaker-level performance, as visualized in Figure 4. Globally, model predictions of depressed mood are now correlated more strongly with the ground truth ratings (Figure 4, top). However, no clear trend of improvement between the baseline model and the personalized network can be observed when looking at subjects individually (Figure 4, center). In contrast, our proposed Hyperformer personalization does not suffer from this problem, increasing or keeping performance for each speaker (Figure 4, bottom) while still improving the accuracy of predictions globally. Finally, Table 3 shows that the output of these simpler personalized models are most strongly correlated with the scores from PHQ-9, HRSD-24, and SEK compared with the other baselines and all hyperformer models, hinting at overfitting on the metadata.

Overall, global performance measurements are ill-suited to further analyze the performance of personalization approaches for depressed mood monitoring on the data at hand, and we will restrict our analysis to speaker-level ρ and put a special focus on participants with MDD (patients) as the group of most interest.

Focusing on speaker-level performance, fine-tuning wav2vec and our proposed personalization approach leads to substantially higher Spearman’s ρ than the FFNN baselines. Moreover, every evaluated hyperformer model configuration outperforms its respective non-personalized fine-tuned wav2vec baseline when analyzed over all subjects. When we disaggregate the results by subject group, these two approaches work better on individuals showing at least some degree of depressive symptomatology (subjects in the patient and subclinical groups), measured in per-speaker ρ.

### Influence of speech content

Following initial work on the dataset and task conducted in Gerczuk et al.,^34^ we further analyze the performance of the best personalized wav2vec model with regard to the content of speech. As can be seen in Table 4, the monitoring of depressed mood works best when basing the deep learning analysis on the answers to mood-related questions. This is in concordance with the previous finding in Gerczuk et al.^34^ and further supported by the acoustic analysis under Exploratory data analysis, where correlations between self-rated mood and paralinguistic speech markers were more pronounced than for the other types of samples. Moreover, because we utilize a wav2vec model pre-trained on German speech recordings, our approach will capture linguistic information about the answers, a phenomenon that has been shown to occur in such models.^51^ Given the nature of the questions, the content of the answers should strongly correlate with the corresponding depressed mood ratings.

**Table 4:** Speech-based results (mean and SD over 5 folds)

	All	Control	Subclinical	Patients
Question	0.192±0.035	0.146±0.057	0.190±0.050	0.231±0.076
Answer	0.435±0.039	0.373±0.071	0.455±0.071	0.467±0.082
Positive thought	0.226±0.028	0.191±0.047	0.199±0.050	0.283±0.020

Performance computed as Spearman’s ρ over each speaker independently and then averaged over all speakers. Grouped by type of audio recording.

It has also been shown that the type of utterance (e.g., answer or question) has an impact on speech emotion recognition performance,^52^ with the hypothesis being that the underlying dialog act constrains the space in which acoustic parameters may vary. In our case, reading out these questions provides no semantic information relevant to a person’s current depressed mood and further restricts the variability of certain depression-related acoustic parameters, such as changes in the rate of speech or number of pauses, to a minimum. Because analyzing these types of speech recordings leads to the overall worst performance, this hypothesis may apply in our case, too.

Most interesting, however, are the spontaneous positive thoughts that participants should record three times at the end of each session. Overall, and quite intuitively, performance on these samples sits between the answers and questions. The free-text nature of these recordings allows changes in speech characteristics, such as the number of pauses, speech rate, and variance of pitch, to manifest more distinctly. Furthermore, because impairment of positive cognition has been shown to be associated with depressive symptomatology,^53^ the ability of study participants to spontaneously formulate positive thoughts could likely be impacted by depressed mood. However, the effects and relationships with self-rated mood differ between experimental groups. When considering recordings of subjects afflicted with MDD (Table 4, patients), speaker-level performance is improved compared with the reading of the questions, while for the subclinical group, no such improvement can be found. Because depression is characterized by negative cognitive biases, rumination, and often a lack of positive biases,^54^ we suspect the effects of depressed mood on positive cognition to be more distinct in the patient group. We plan to perform a more thorough investigation of this suspicion in future work.

### Ablation of chosen metadata

We performed an ablation study on the choice of metadata, which we utilized to condition the weight generation of adapter modules in the hyperformer architecture. The best-performing configuration takes in the complete set of chosen meta-information, consisting of demographic and medication information, the results of the TIPI, the SEK scores for mood regulation, and three depression questionnaires (PHQ, HRSD, and BDI). We trained models for each of the members of this set and additionally combined the scores of the depression questionnaires. We join demographic information and medication together because the latter only contains two items, and both can be considered general information that is available before the first psychological screening. The results can be found in the lower half of Table 2. Computed over all speakers, utilizing only depression questionnaires matches performance with the whole set (0.371 vs. 0.373) but with a slight hit to stability. On the other hand, restricting the conditioning embeddings to demographic information and details about current medication leads to the worst performance overall and for each subject group. Compared with more comprehensive diagnostic information, such as that found by the HRSD, the demographic information might be insufficient to further explain intra-individual mood variations. We observe differing behavior when looking at the subject groups individually. While mood recognition sees the largest benefit from personalization based on the depression tests for the patients, again matching performance with taking in all meta-information, individuals in the subclinical group do not benefit as much. Because these subjects are not diagnosed with MDD but only show some subset of depressive symptomatology to moderate degrees, the scores from depression tests might not provide sufficient information for the analysis of how depressed mood is reflected in their speech. Interestingly, personalization based on the TIPI leads to a substantial speaker-level performance boost in both groups, more than the inclusion of depression tests in the subclinical group. As a very brief 10-item inventory, its questions focus on measuring the Big Five personality dimensions: openness, conscientiousness, extraversion, agreeableness, and neuroticism. A considerable amount of research has been conducted on the relationship between personality traits and depressed mood or depression,^55^^,^^56^ e.g., linking neuroticism to depression severity and depression proneness^57^ or showing that high neuroticism and low extraversion correlate with more negative mood and higher mood variations.^58^ Highly relevant to our study, Duberstein and Heisel^59^ showed that high neuroticism leads to overreporting depressed mood, while more openness increases the tendency toward underreporting. In this way, the TIPI seems to be informative for modeling inter-individual differences in the relationship between the self-assessment of depressed mood and its physiological influence on speech.

### Fairness

As a last step, we examine our models in terms of fairness across different individuals. There are different forms of individual fairness depending on the specific problem: most works require “similar users to receive similar outcomes,”^60^ a formulation most suited to use cases where the model is evaluated on each individual once (e.g., in credit score assessment). However, in our case, we have an artificial intelligence (AI) system that continually tracks the mood of each individual and is thus evaluated on instances of them several times. We therefore adopt the requirement that the system ideally achieves an equal speaker-level performance across individuals.

One principled way of measuring the discrepancy across different speakers is the Gini index, which has also been used before for individual fairness in depression monitoring.^12^ Primarily used in economics, it is a measure of inequality, with higher values indicating that utility (e.g., income) is largely concentrated on a few individuals. In our case, this utility is simply speaker-level performance. Thus, the Gini index measures how that performance is distributed across different patients: low values indicate an “egalitarian” setup, where the system performs equally well for most or all speakers, whereas higher values show an imbalance toward a subset of them.

Results are shown in Table 5, where we focused on patients as the group of highest interest. The baseline system shows moderate levels of inequality, with an average Gini index over all 5 folds of 0.421. All hyperformer models are able to improve on that, with personalization relying on depression test scores reaching a best performance of 0.325. This shows how introducing personal information, even in limited form, can provide a system whose performance is more equally distributed across users.

**Table 5:** Individual fairness for patients is computed as the Gini index over speaker-level Spearman’s ρ (mean and SD over 5 folds; lower → more equal), including the number of winners (patients for whom performance improved) and losers (patients for whom performance worsened) as a result of personalization

Model	Gini index	Winners	Losers
Finetuned wav2vec	0.421±0.170	–	–
Hyperformer all	0.369±0.148	32(132%)	16(27%)
Hyperformer PHQ-9	0.354±0.156	32(156%)	16(24%)
Hyperformer HRSD	0.348±0.111	$33^{*}\;\left(126\%\right)$	$15^{*}\;\left(35\%\right)$
Hyperformer BDI	0.362±0.131	31(120%)	17(34%)
Hyperformer depression tests	$0.325^{*}\pm 0.157$	31(157%)	17(26%)
Hyperformer demographics + medication	0.401±0.184	31(128%)	17(43%)
Hyperformer SEK	0.356±0.126	31(120%)	17(34%)
Hyperformer personality	0.349±0.156	29(180%)	19(23%)

Asterisks (∗) denote the best result for each metric.

Furthermore, we are interested in measuring how our proposed personalization impacts speaker-level performance across individuals. This can be useful in cases where a personalized model is proposed as an alternative to a baseline model already used for monitoring patients; “upgrading” to a better model must be justified by improved outcomes for all users. By conceptualizing personalization as an intervention (targeted at a diagnostic method for depression), we measured how many individuals benefitted from this intervention (i.e., their performance improved; “winners”) vs. those whose performance worsened (“losers”). All personalized models improved the performance of the majority (>60%) of users. This showcases how most users will benefit from personalized monitoring of their mood. Importantly, “winners” see a much bigger percent improvement in their performance than “losers” see a drop, meaning that, even for the cases where personalization fails, the deterioration is not nearly as great.

## Discussion

Previous speech-based research on depression has been largely geared toward the detection of disease status.^19^ Typically, this boils down to either the classification of an individual into a patient or control group or the prediction (e.g., regression) of a depression-related scale, such as PHQ-9 score. An alternative line of work pursues the more granular characterization of depression tracking mood states or depression scales. For example, Karam et al.^22^ monitor telephone conversations of bipolar disorder patients over time and classify depression states derived from the HRSD and Young Mania Rating Scale (YMRS). Most similar to our work, Song et al.^25^ make personalized predictions of Discrete Analog Mood Scale (DAMS) items from prompted EMAs of Japanese participants. The work presented in this paper falls into this latter line. From a clinical perspective, exploring affective dynamics may allow the assessment of subtypes of depression.^61^ Further, high mean negative affect is associated with depression risk,^62^ and instability in affective states may be an indicator of depression and anxiety.^63^ Moreover, deficits in emotion regulation are a stable predictor of depressive symptoms.^64^ In this context, utilizing an automated recognition of depressed mood could give subjects a nuanced way of tracking their emotional state and indicating the success of affective regulatory strategies.

However, approaches that model depressed mood on the population level lack the capability to adapt to differences in mood regulation between patients,^33^ necessitating the development of personalization strategies. In this paper, we introduced a strategy for personalization of large-scale speech foundation models for the automatic monitoring of depressed mood to account for this issue. Compared with previous works on the topic,^25^^,^^34^ our approach does not rely on an enrollment phase but, rather, works solely based on available metadata. We utilize hyperformer adapters,^37^ introduced for multitask language learning, to efficiently inject subject-specific metadata into large wav2vec transformer models. Through a structured evaluation, we showed that the approach improves the recognition of intra-individual mood variations. Furthermore, our results indicate the importance of incorporating meta-information throughout the whole architecture of a neural network instead of a singular point of multimodal fusion. Finally, the ablation of included metadata showed that the scores of items from the PHQ-9 had the largest impact on performance. However, the TIPI, which, as a personality questionnaire, does not measure depressive symptom severity, provides a similar boost in performance.

The work presented here is affected by some limitations. First of all, the relatively short-term nature of the dataset prevents us from analyzing the impact of our proposed metadata-based personalization strategy over larger timespans. While symptomatology is relatively stable over a short 2-week period, individuals afflicted with MDD can experience remission, relapse, or re-occurring depressive episodes of varying severity during the disease. By only utilizing diagnostic information obtained in the baseline assessment, we do not explicitly model this drift in depression-related speaker state. Future work should investigate the impacts on the performance of our and similar approaches when faced with this drift. Furthermore, after a post hoc inspection of our experimental results, we ascertained that the two subjects with the lowest speaker-level performance appear to be using the scale in the opposite way as instructed. Their depressed mood self-ratings were higher when their tone of voice and sentiment appears (to the authors) positive and lower when their tone appears more negative/depressed. However, it was impossible to establish contact with these participants after the study had ended and thus verify our hypothesis. Because such glitches are inevitable in real-life studies, we decided to include these participants in our results with their original ratings, subject to the caveat that these scales might be, in truth, inverted and therefore could have had a negative impact on the model training and performance. Finally, because our data were collected “in the wild” by participants themselves, potential biases might be introduced in the data collection process, such as some participants recording data in different locations depending on their mood. Given that speech models, including w2v2, are affected by background noise,^65^ this may inadvertently introduce some bias into our results. However, we do not expect this bias to be large for most participants, given the large amount of samples they collected (μ=355.1).

There exist a couple of possible research directions that should be explored. While our approach considers conditioning only based on subject-specific metadata, an extension to voice characteristics should be investigated in future work. An intuitive extension of our personalization framework could see the inclusion of speaker embeddings computed over external voice samples as an additional input. Similarly, our approach could be transformed into a few-shot personalization strategy by the inclusion of baseline speech samples injected through the same means as the metadata. While the notion of a “neutral” baseline sample, as used, for example, in Triantafyllopoulos et al.^66^ for speech emotion recognition, is not directly transferable to the monitoring of depressed mood, reappraisal statements combined with diagnostic scores such as the SEK could further inform how affective states are expressed in the voice of individual subjects. Finally, the linguistic content of the prompted positive thoughts should be analyzed and incorporated more directly; e.g., through different fusion approaches with large, pre-trained language models.

## Experimental procedures

### Resource availability

#### Lead contact

Further information and requests for resources should be directed to and will be considered by the lead contact, Maurice Gerczuk (maurice.gerczuk@uni-a.de).

#### Materials availability

This study did not generate new unique reagents.

### Zero-shot personalization through metadata-conditioned hyperformers

In order to achieve a zero-shot personalization of depressed mood recognition based on diagnostic and demographic meta-information, we adapt the methodologies of Houlsby et al.^36^ and Karimi Mahabadi et al.^37^ for multitask learning of natural language processing (NLP) problems. The former introduced the notion of adapter modules to the transformer architecture, while the latter experimented with utilizing hypernetworks to generate the weights of these adapters based on task embeddings. These adapter modules become the means through which we inject personalized information into large-scale, pre-trained speech foundation models. In the following, we first describe transformer adapter modules and later our proposed personalized weight generation process based on subject metadata, including demographic and diagnostic information.

Adapters are small, additional neural network components that are inserted within the original structure of the pre-trained neural network, keeping the base model’s parameters intact.^68^ During transfer learning, all of the base model’s parameters are frozen, and only the weights of the added adapter modules are trained. Compared with traditional transfer learning strategies, adapters are more parameter efficient than full-fine-tuning and have been shown to perform competitively.^36^

**Figure 5:**
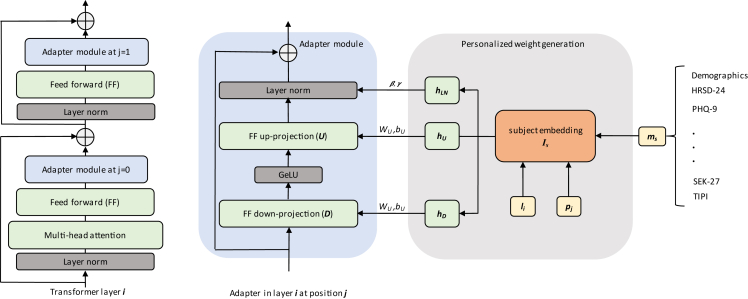
Architecture of personalized transformer Visualization of our proposed zero-shot personalization of transformer-based speech foundation models. The left shows where adapter modules for personalization are inserted into a single layer of the wav2vec encoder. An overview of the weight generation process for these adapter modules is shown on the right. A vector of subject metadata is embedded together with learned layer and position embeddings resulting in $I_{s}$. This embedding then conditions three hypernetworks to generate the weights for the down- and upsampling feedforward layers and the parameters for the LN, respectively.

We outfit the baseline wav2vec model with adapter modules, as outlined for the t5 language transformer by Houlsby et al.^36^ In each encoder layer, adapters are inserted after the attention and feedforward blocks but before the additive skip connections. Such a transformer layer is displayed on the left of Figure 5. Each adapter consists of a feedforward down-projection (D), Gaussian Error Linear Unit (GeLU)^69^ non-linearity, up-projection (U), and layer normalization (LN). Furthermore, an additive skip connection bypasses these components. We denote the weight matrices and biases of the down- and up-projection as $W_{D}\in R^{h\times d}$, $b_{D}\in R^{d}$, and $W_{U}\in R^{d\times h},b_{U}\in R^{h}$, with h being the dimension of the input x and d the size after down-projection. The output of the adapter d is then computed from the input hidden state x as^70^

(Equation 1) A(x)=LN(U(GeLU(D(x))))+x.

The LN is parameterized by $\beta \in R^{h}$ and $\gamma \in R^{h}$:

(Equation 2) $$\mathrm{LN}\left(x\right)=\frac{x-\mu }{\sigma }⊙\gamma +\beta \text{.}$$

Mean (μ) and standard deviation (σ) are computed across the elements of x, and β and γ are learned during training. In our personalized machine learning setting, the adapter in transformer layer l for subject S is parameterized by $D_{S}$, $U_{S}$, and $LN_{S}$, each with distinct weights and biases.

While the above approach applies to a time-dependent personalization strategy that uses a certain number of data points from the target subject, it needs to be adjusted to achieve zero-shot personalization. We utilize the concept of hypernetworks to generate the parameters for the subject conditional adapter modules, as presented in^37^ for multi-task language modeling. The networks are conditioned on subject embeddings $I_{S}$ which we project through a network $h_{I}$ from metadata vectors $m_{S}$. In this way, they learn to derive shared information between individual subjects solely based on diagnostic and demographic information. To cut down on the number of trainable parameters, we further apply a variation of this concept in which the parameters of each adapter module throughout the whole Transformer encoder are generated by the same hypernetworks.^37^ This is achieved by concatenating layer and position (after attention or after feedforward) embeddings ($l_{i},i\in \left\{0,\ldots ,23\right\}$, $p_{j},j\in \left\{0,1\right\}$) with the subject metadata vector $m_{S}$ before projection through $h_{I}$:

(Equation 3) $$I_{S}=h_{I}\left(m_{S},l_{i},p_{j}\right)\text{.}$$

While $m_{S}$ is a fixed input parameter, $l_{i}$ and $p_{j}$ are generated through embedding matrices learned end-to-end via backpropagation. The projection network $h_{I}$ consists of a fully connected (FC) layer with Rectified Linear Unit (ReLU) activation and a linear FC layer. The weight generation process for the adapter and LN in encoder layer i for position j is visualised on the right of Figure 5. Parameters for down- and up-projection layers $D_{S}^{i,j}$ and $U_{S}^{i,j}$ are produced by the hypernetworks $h_{D}$ and $h_{U}$:

(Equation 4) $$\left(D_{S}^{i,j},U_{S}^{i,j}\right):=\left(h_{D}\left(I_{S}\right),h_{U}\left(I_{S}\right)\right)=\left(W^{D},W^{U}\right)I_{S}\text{,}$$

with $I_{S}$ defined as in Equation 3. Given input dimension h, bottleneck size d, and subject embedding dimension s, $h_{D}$ and $h_{U}$ have to generate both weights and biases for the down- and up-projection layers, resulting in weight matrices $W^{D}\in R^{h\times \left(d+1\right))\times s}$ and $W^{U}\in R^{d\times \left(h+1\right)\times s}$. Similarly, $h_{\mathrm{LN}}$ computes $\beta_{S}^{i,j}\in R^{h}$ and $\gamma_{S}^{i,j}\in R^{h}$ via $W^{\beta }\in R^{s\times h}$ and $W^{\gamma }\in R^{s\times h}$:

(Equation 5) $$\left(\beta_{S}^{i,j},\gamma_{S}^{i,j}\right):=h_{\mathrm{LN}}\left(I_{S}\right)=\left(W^{\beta },W^{\gamma }\right)I_{S}\text{.}$$

### Model training

The personalized hyperformer models are initialized from the finetuned wav2vec baseline models and outfitted with adapter modules and their weight-generating hypernetworks. As reported in Karimi Mahabadi et al.,^37^ the hypernetworks and transformer base model cannot be trained conjointly in a stable fashion. Therefore, we freeze all parameters except for the added hypernetworks and the final fully connected prediction layer. Analogous to the baseline wav2vec models, we train the personalized hyperformer with a batch size of 16 and a learning rate of $3e^{-4}$ utilising AdamW^71^ with learning rate warmup for a maximum of 10 epochs. Most of the models reach their best performance on the validation set before the fifth epoch. We choose this best checkpoint for evaluation on the test set.

### Baselines

We compare our proposed zero-shot personalization strategy against a set of baselines, consisting of non-personalized approaches and a simple metadata personalization via subject embeddings.

#### FFNN

The first baseline is an FFNN with 3 hidden fully connected layers with ReLU activation function trained on pre-extracted features. We implement two versions of this network, one trained with the eGeMAPS^72^ of audio functionals extracted with openSMILE^73^ and the other on wav2vec embeddings extracted from the same model, which we fine-tune and personalize for our proposed approach. For the eGeMAPS model, we choose a hidden size of 30 for all three layers, while the wav2vec embeddings have a larger dimensionality, motivating an increased hidden size of 256. We choose AdamW as optimizer with a learning rate of $1e^{-4}$ and train the models in batches of 128 samples for a maximum of 100 epochs. For these FFNN models, we further experiment with a simplified metadata personalization by embedding the same subject vectors as used in the best-performing hyperformer configuration through a linear projection layer and performing elementwise addition with the output of the model backbone (after the second hidden layer).^25^

#### Fine-tuned wav2vec

We fine-tune a pre-trained wav2vec model as an additional baseline which we later further adapt with our zero-shot personalization method. It, therefore, lets us analyze the performance gains achieved through the personalization strategy most directly. For this baseline, we utilize the pre-trained German XLSR-53 large model obtained from the huggingface hub (https://huggingface.co/jonatasgrosman/wav2vec2-large-xlsr-53-german) and freeze the weights of the convolutional feature extractor, only fine-tuning the transformer. The model is trained with a batch size of 16 and a learning rate of $3e^{-4}$ for a maximum of 10 epochs. We follow the best practices for training wav2vec; i.e., we utilize the AdamW optimizer and perform a learning rate warmup at the beginning of the training.

### Evaluation

We evaluate all models via a speaker-independent 5-fold cross-validation where we additionally split a portion of each training dataset’s speakers to form a validation set. In this setup, every speaker appears exactly once in one of the 5 folds’ test sets. For all considered approaches, we choose the best model for evaluation on the test set based on its performance on each fold’s speaker-independent validation set, measured in the global Spearman’s correlation coefficient.

## Data Availability

The utilized data are not publicly available because they contain sensitive personal information. Access for the purposes of review can be requested by contacting the lead author. All original code is available at https://github.com/mauricege/HyperPersonalisation. A snapshot of the repository at the time of publication has been uploaded to Zenodo: https://doi.org/10.5281/zenodo.8328092.^67^
